# Utilization of prospective, pharmacist-driven restriction criteria to improve appropriateness of empiric intravenous vancomycin use

**DOI:** 10.1017/ash.2026.10808

**Published:** 2026-07-23

**Authors:** Kinsey McClure Johannemann, Mark Jumper, Blain Thayer, Djeunou Tchamba

**Affiliations:** 1 Piedmont Medical Center, USA; 2 University of New Mexico - Albuquerque: The University of New Mexico, USA

## Abstract

**Objective:**

This objective of this study is to evaluate the impact of a prospective, pharmacist-driven restriction protocol on the appropriateness of IV vancomycin use in a community hospital setting.

**Methods:**

This single-health system, multi-site, quasi-experimental study compared pre-intervention (January–March 2024) and post-intervention (January–March 2025) groups. The intervention was a mandatory pharmacist-driven protocol requiring review of every empiric vancomycin order against pre-defined, evidence-based criteria. Inappropriate orders were proactively discontinued, and providers were notified with supporting rationale. The primary outcome was the percentage of appropriate empiric vancomycin orders, assessed by chart review of 100 random patients per group. Key secondary outcomes included vancomycin days of therapy per 1,000 patient days and trough levels ordered per 1,000 patient days.

**Results:**

Analysis of 200 patients (100 per group) showed that the appropriateness of empiric IV vancomycin orders significantly increased from 55% pre-intervention to 97% post-intervention (P < .001). Days of therapy per 1,000 patient days decreased from 69.3 to 64.2 (P < .041), and vancomycin trough levels ordered per 1,000 patient days decreased by 41% (52.3 vs. 31.1, P < .001). Crucially, no significant differences were observed in 30-day mortality, suggesting the improvements were achieved without compromising patient safety in patients who received vancomycin.

**Conclusions:**

Implementation of a prospective, pharmacist-driven empiric IV vancomycin restriction protocol offers a practical, effective strategy for institutions seeking to increase appropriateness of empiric IV vancomycin orders.

## Introduction

Vancomycin is first-line therapy against many methicillin-resistant *Staphylococcal* spp. infections in hospitalized patients, making it one of the most-utilized antibiotics in the inpatient setting. Despite the introduction of new anti-MRSA agents, vancomycin remains the preferred agent in many institutions due to its relatively low cost, extensive clinical data, and widely-available pharmacokinetic monitoring. Although vancomycin is recommended empirically in several infections, such as central nervous system infections^1–3^ or endocarditis,^4–6^ its initial use is not warranted for many disease states. Recommendations for vancomycin stewardship date back to 1995, when rapid increases in the incidence of vancomycin-resistant *Enterococcus* isolation prompted the Hospital Infection Control Practices Advisory Committee (HICPAC) to publish recommendations to prevent and control the spread of vancomycin resistance.^7^ Its first recommendation is to promote prudent vancomycin use by clinicians by developing institutional guidelines for appropriate use.^7^ Even now, after decades of promoting vancomycin stewardship, it is estimated that up to 67%^8–13^ of total IV vancomycin use is unnecessary, with critical clinical condition and failure to de-escalate antimicrobials as driving risk factors for inappropriate use.^7^

Prudent use of vancomycin is not only imperative to decreasing the spread of vancomycin-resistant organisms, but also to minimizing adverse events associated with its use such as nephrotoxicity and infusion-related reactions. Despite the introduction of new anti-MRSA agents, excessive vancomycin use strains already-limited healthcare resources. Beyond the cost of preparation, administration, and the drug itself, one must also consider the costs of pharmacokinetic monitoring^14–16^ (including pharmacist time, lab costs, etc.) and mitigating any adverse events^16^ (increased lab monitoring, prolonged hospitalization, etc.).

At our institution, vancomycin is the second-most utilized antimicrobial, surpassed only by ceftriaxone. Prior to this study, there were no institutional pathways or restriction criteria to guide its use beyond general education among providers and pharmacists. In October 2024, our hospital implemented several IV fluid preservation strategies due to a national fluid shortage caused by Hurricane Helene. Since our pharmacy team historically reported anecdotal overuse of vancomycin, this prompted the creation of institutional empiric vancomycin restriction criteria to not only reduce IV fluid use, but also to decrease unnecessary vancomycin use in general. The objective of this study is to evaluate the impact of a prospective, pharmacist-driven restriction protocol on the appropriateness of IV vancomycin use in a community hospital setting.

## Methods

### Study design

This single health-center, multisite, quasi-experimental study was approved as an internal quality improvement project by Piedmont Medical Center, and therefore exempted from IRB review. Empiric vancomycin restriction criteria were developed and approved by the institution’s antimicrobial stewardship team (AST), which consists of two infectious diseases pharmacists, one infectious diseases physician, two advanced practice clinicians, one microbiologist, and one infection prevention specialist. These pharmacist-driven empiric vancomycin restriction criteria were implemented in October 2024. This study was divided into two 3-month periods, with the preintervention period spanning from January 1^st^ to March 30^th^, 2024 and the postintervention period spanning January 1^st^ to March 30^th^ of 2025, allowing for a sufficient washout period after protocol implementation.

The primary outcome of this study was the percentage appropriate empiric vancomycin orders before and after the implementation of a pharmacy-driven restriction protocol. A subset of 100 random patients from each group were selected to be analyzed via detailed chart review to accommodate manpower limitations. This selection was made via random number generation in Excel (Microsoft Corporation, Redmond WA). The final determination of appropriate versus inappropriate for study purposes was made by the AST pharmacist via chart review and was strictly based on protocol adherence. Secondary outcomes included DOT/1,000 patient days for IV vancomycin, total IV vancomycin acquisition cost, and total number of vancomycin levels (trough and random) ordered per 1,000 patient days. Patient-specific secondary outcomes were also measured in the two cohorts and included total days of inpatient IV vancomycin therapy, outcome of empiric therapy, hospital length of stay, acute kidney injury (as defined in KDIGO guidelines), 30-day mortality, and suspected infusion reactions.^17^

### Patient population

Patients were identified retrospectively from the electronic health record via a report of all initial pharmacy vancomycin dosing consults. Patients were included in this study if they were aged ≥18 years, admitted as an inpatient for ≥48 hours after the dosing consult was placed, and had vancomycin ordered as empiric therapy. Empiric therapy was defined as being ordered prior to obtaining culture results. Patients who received one-time doses in the emergency department or as surgical prophylaxis, pregnant patients, and patients with vancomycin being continued as a home medication were excluded from this study.

### Intervention

**Figure 1.**
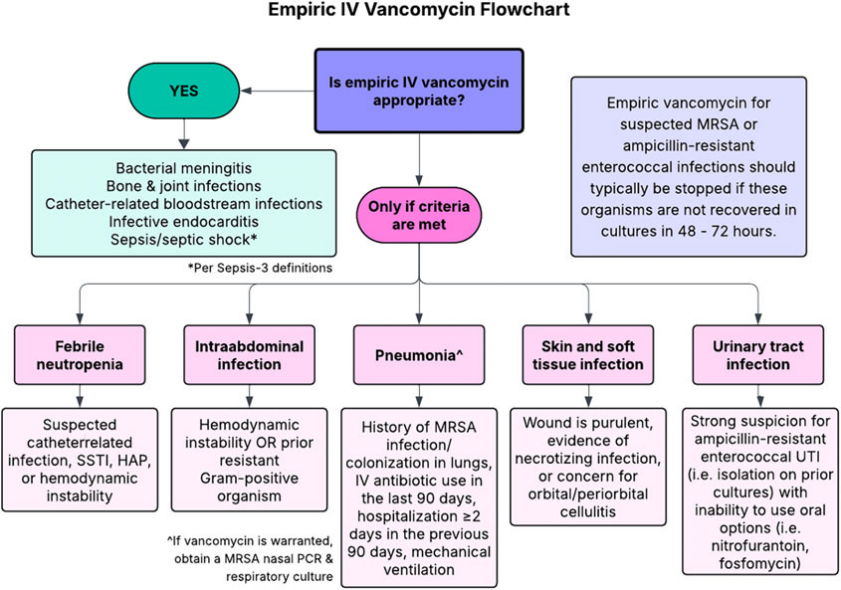
Figure 1 long description. A flowchart summarizing criteria for empiric IV vancomycin use. It guides decision-making based on infection type and patient-specific risk factors.

This pharmacy-driven empiric vancomycin restriction criteria and protocol was implemented across two community hospitals in a single health system. Per this protocol, pharmacists reviewed each vancomycin order upon pharmacokinetic dosing consult to verify that it is appropriate per the restriction criteria. Pharmacokinetic dosing consults are automatically reflexed for all vancomycin orders outside of one-time doses in the emergency department and surgical prophylaxis. Appropriate empiric indications were formulated based on HICPAC recommendations for appropriate vancomycin use, various international society guidelines, and expert opinion.^1–7,18–27^ These indications can be viewed in Table 1 as well as Figure 1, which was distributed to both pharmacists and providers for educational purposes. If vancomycin was deemed appropriate, the pharmacist would proceed with the pharmacokinetic consult. If inappropriate, pharmacists would automatically discontinue the vancomycin pharmacokinetic consult and notify the provider of the discontinuation with supporting rationale. If the provider responded with the desire to override the protocol and proceed with vancomycin use, the pharmacist would document their rationale and notify the AST pharmacist for review. During daily pharmacokinetic consult follow-up, pharmacists would assess the need to continue empiric vancomycin therapy by 48–72 hours. If no clinical evidence (eg, cultures, imaging, vitals) was present of a Gram-positive infection warranting vancomycin use was present, the pharmacist would contact the provider with an assessment of the patient and strongly recommend vancomycin discontinuation. If the provider countered this request with inappropriate rationale, the recommendation would be escalated to the AST pharmacist for review.

**Table 1.** Empiric IV vancomycin restriction criteria Table 1 long description.

Appropriate empiric indications		
Bacterial meningitis		Infective endocarditis
Bone and joint infections		Sepsis^*^
Catheter-related bloodstream infections		Septic shock^*^

Possibly appropriate indications if additional criteria met		
Febrile neutropenia	-Suspected catheter-related infection, SSTI, HAP, or hemodynamic instability	
Intraabdominal infection	-Hemodynamic instability -Prior resistant Gram-positive organism	
Pneumonia	-History of MRSA pulmonary infection or colonization -IV antibiotic use in the last 90 days -Hospitalization ≥2 days in the previous 90 days -Mechanical ventilation	
Skin and soft-tissue infection	-Purulent or necrotizing infection -Orbital and periorbital cellulitis	
Urinary tract infection	-Strong suspicion for ampicillin-resistant enterococcal UTI (i.e. isolation on prior cultures) with inability to use oral options (i.e. nitrofurantoin, fosfomycin)	

* Per Sepsis-3 definitions. HAP, hospital-acquired pneumonia; MRSA, methicillin-resistant *Staphylococcus aureus*; SSTI, skin and soft tissue infection; UTI, urinary tract infection.

### Statistical analysis

Descriptive and inferential statistical analyses were conducted. Continuous data were presented as either means with standard deviations or medians with interquartile ranges (IQR) and were analyzed with a Student’s *t*-test or Mann–Whitney *U* test, as appropriate. Categorical data were presented as counts with percentages and analyzed with χ^2^ or Fisher’s exact test, as appropriate. Two-tailed *P*-values of less than .05 were considered statistically significant. Relative risks were calculated to compare outcomes in the postintervention group with those in the preintervention group. Relative risks were not calculated for continuous outcomes or for mutually exclusive empiric therapy disposition categories, and a continuity correction was used for infusion reactions. Data was analyzed using SPSS v29.0.1.1 (IBM, Armonk, NY).

## Results

A total of 200 patients were evaluated in this study, with 100 in the preintervention group and 100 in the postintervention group. There were no significant differences between age, gender, history of MRSA infection, recent hospitalization, or ICU admission between groups (Table 2). In the preintervention group, the most common IV vancomycin indications were skin and soft-tissue infections, pneumonia, and sepsis/septic shock. In the postintervention group, the most common indications were bone and joint infections, pneumonia, and sepsis/septic shock (Table 2). The appropriateness of IV vancomycin orders increased from 55% in the preintervention group to 97% postintervention (RR 1.76, 95% CI 1.47–2.11; Table 3). Of those placed on vancomycin, the median inpatient length of therapy was 4 days both pre and postintervention. Empiric IV vancomycin was either continued as definitive therapy or empiric therapy remained appropriate for 18% of the preintervention group and 16% of the postintervention group (Table 3).

**Table 2.** Baseline characteristics Table 2 long description.

Characteristic	Pre-intervention group (n = 100)	Post-intervention group (n = 100)	*P*-value
Age in years, *median (IQR)*	65 (53–76)	66 (54–75)	.915
Male gender, n (%)	45	44	1.000
History of MRSA infection, n (%)	7	7	1.000
Recent hospitalization, n (%)	40	34	.464
ICU during admission, n (%)	28	35	.361
Ordering indication, n (%)			
BSI	0	0	1.000
BJI	9	23	.011
CNS	0	5	.059
FN	1	1	1.000
IE	1	0	1.000
IAI	1	0	1.000
PNA	29	23	.420
Sepsis/septic shock	25	29	.641
SSTI	32	19	.051
UTI	1	0	1.000
Other	1	0	1.000

History of MRSA infection was defined as MRSA isolated in a culture from any site within the past 12 months. Recent hospitalization was defined as a history of hospitalization >48 hours in previous 3 months. Ordering indication determined via statements in provider progress notes. BSI, bloodstream infection; BJI, bone and joint infection; CNS, central nervous system; FN, febrile neutropenia; HAP, hospital-acquired pneumonia; IE, infective endocarditis; IAI, intraabdominal infection; ICU, intensive care unit; MRSA, methicillin-resistant *Staphylococcus aureus*; PNA, pneumonia; SSTI, skin and soft tissue infection; UTI, urinary tract infection.

**Table 3.** Primary and secondary outcomes Table 3 long description.

Outcome	Pre-intervention group (n = 100)	Post-intervention group (n = 100)	*P*-value
Appropriate order, n (%)	55 (55)	97 (97)	**<.001**
Days of inpatient vancomycin therapy, n (IQR)	4 (3–6)	4 (2–7)	.609
Outcome of empiric therapy			
Continued as definitive therapy	3	8	.221
Continued empirically-appropriate	15	8	.154
Continued empirically-not appropriate	22	31	.177
Stopped by 48–72 hours	60	50	.169
Switched to alternative IV anti-MRSA drug	0	3	.083
AKI	17	22	.476
Infusion reaction	3	0	.246
30-day mortality	8	11	.631

Outcome	Pre-intervention period	Post-intervention period	
DOT/1,000 patient days	69.3	64.2	**<.041**
Vancomycin levels ordered/1,000 patient days	52.3	31.1	**<.001**

DOT, days of therapy; MRSA, methicillin-resistant *Staphylococcus aureus.*

*Note*: appropriateness of vancomycin being continued empirically was determined by the availability of culture data and other laboratory/diagnostic testing pertaining to the disease state for which vancomycin was ordered.

No significant difference in the incidence of AKI (RR 1.29, 95% CI 0.73–2.29), infusion reactions (RR 0.14, 95% CI 0.01–2.73), or 30-day mortality (RR 1.38, 95% CI 0.58–3.27) were identified between groups (Table 3). Of the 50 patients who had vancomycin stopped at 42–72 hours postintervention, only one patient went on to have an anti-MRSA agent restarted later in the hospitalization. However, the vancomycin was restarted empirically for a suspected hospital-associated infection and was quickly discontinued, as no resistant Gram-positive organisms were isolated via culture.

When data were combined, the two sites included in this study showed a decrease in total IV vancomycin use after the intervention was implemented as evidenced by the total IV vancomycin DOT per 1,000 patient days decreasing from 69.3 preintervention to 64.2 postintervention (*P* < .04). The total IV vancomycin acquisition cost decreased from $15,393 in the preintervention period to $13,790 in the postintervention period. In addition to a decrease in drug use, total vancomycin levels ordered per 1,000 patient days decreased from 52.3 preintervention to 31.1 postintervention (*P* < .001).

## Discussion

In this quasi-experimental study conducted across two community hospitals, implementation of a pharmacist-driven empiric IV vancomycin restriction protocol was associated with a 42% (*P* < .001) increase in appropriateness of vancomycin order indications without compromising patient safety. Additionally, the intervention was associated with a reduction in total IV vancomycin use, pharmacokinetic monitoring burden, and resource utilization, while clinical outcomes such as 30-day mortality remained unchanged.

Prior studies examining the incidence of inappropriate empiric vancomycin use report rates as low as 10.9% in specialized units (eg, cardiac surgery units, hematology/oncology) to 79% in institutionwide evaluations, which is consistent with our preintervention findings and reinforce the need for increased stewardship efforts.^10,11,28,29^ The majority of published interventions on vancomycin restriction criteria require a multidisciplinary antimicrobial stewardship program, infectious diseases physician involvement, or process changes in the electronic medical record system at the order-entry.^29–36^ In contrast, our study incorporated restriction criteria into an existing pharmacist workflow, which may be more feasible in institutions with limited resources. Pharmacists were given the autonomy to assess orders upon receipt of the pharmacokinetic consult and influence clinical decisions upstream to optimize IV vancomycin use early in therapy. The high appropriateness rate postintervention suggests strong adherence to this process among frontline pharmacists and general acceptance among providers, though formal provider acceptance rates were not captured in this study. The jump in appropriate orders from 55% to 97% highlights that targeted, practical interventions—such as front-end review, prescriber education, and protocol alignment—can influence prescribing behavior quickly and sustainably.

Pharmacists at our institution evaluate all patients receiving vancomycin daily, so appropriate use was further reinforced by prompting discontinuation in patients without evidence of Gram-positive infections at 48–72 hours. Early discontinuation rates were already high in the preintervention group, which likely limited how much further improvement could be detected. This may also indicate the presence of strong stewardship habits that the intervention reinforced rather than introduced.

This intervention did not appear to compromise patient safety, as evidenced by the high rate of sustained vancomycin discontinuation at 48–72 hours without need for re-initiation. Only one patient required re-initiation of anti-MRSA therapy after initial discontinuation, and even then, vancomycin was quickly stopped again after culture results became available. Additionally, no significant changes in 30-day all-cause mortality were observed, further supporting the safety of early discontinuation in the absence of microbiologic justification. Other safety outcomes like AKI and infusion reactions did not differ significantly between the two periods, which is not surprising considering that there was no significant difference in the days of inpatient vancomycin use between groups. It is also worth noting that the study was not powered to detect changes in secondary outcomes, so a lack of statistical significance here is not unexpected.

On a systemwide level, we observed a 7.4% decrease in overall IV vancomycin use (DOT/1,000 patient days) and a 41% reduction in vancomycin levels ordered. These findings suggest a decreased need for pharmacokinetic monitoring, which has implications for pharmacist workload, nursing resources, and lab costs. Previous studies have shown the significant time and resource investment required for routine vancomycin therapeutic drug monitoring,^14–16^ and our results suggest that this burden can be meaningfully reduced through front-end stewardship interventions.

This study adds to the limited body of literature on pharmacist-driven antimicrobial restriction in community hospital settings. Key strengths include a pragmatic design, the use of standardized and evidence-based criteria, and implementation across multiple hospital sites. The robust postintervention improvement and lack of adverse outcomes further support the feasibility and effectiveness of this approach. However, this study has several limitations as well. First, the retrospective design introduces the potential for documentation bias. Even though there were no significant changes in stewardship activities that were likely to impact vancomycin use, there is still a risk of bias due to factors like physician turnover or unmeasured differences in the pre and postintervention populations. Second, the study was limited to 100 randomly selected patients per period, which may not fully capture institutional trends. Although the protocol required pharmacists to communicate with providers in cases of vancomycin being rejected, we did not formally evaluate provider acceptance rates or resistance to pharmacist recommendations. Because of this, we were unable to formally monitor outcomes in patients who had vancomycin ordered and rejected due to failure to meet restriction criteria. Lastly, the study was conducted within a single health system, which may limit generalizability to other institutions with differing staffing models or patient populations.

Future directions for this research may include assessments of this protocol’s long-term impact on institutional resistance patterns and a formal cost analysis. Additionally, this type of intervention could be applied to additional overused antibiotics. It would also be valuable to assess provider response rates to identify areas for targeted education.

Stewardship of vancomycin is imperative to preserve its clinical effectiveness, minimize antibiotic resistance, and reduce adverse effects in individual patients. A pharmacist-driven restriction protocol significantly improved the appropriateness of empiric vancomycin orders across two community hospitals, with associated reductions in drug use and monitoring. These improvements were achieved without negatively impacting clinical outcomes in patients who received vancomycin. These findings highlight the clinical and operational value of empowering pharmacists to serve as front-line antimicrobial stewards, and this model may serve as a practical intervention to influence prescribing patterns in settings with limited infrastructure or infectious diseases coverage.

## Research transparency and reproducibility

Data is not publicly available.

## Figure 1. Long description

A flowchart summarizing criteria for empiric IV vancomycin use. It guides decision-making based on infection type and patient-specific risk factors. The flowchart starts with the question Is empiric IV vancomycin appropriate? If yes, it leads to conditions like bacterial meningitis, bone and joint infections, catheter-related bloodstream infections, infective endocarditis, and sepsis/septic shock. For febrile neutropenia, it considers suspected catheter-related infection, skin and soft tissue infection, hospital-acquired pneumonia, or hemodynamic instability. For intraabdominal infection, it looks at hemodynamic instability or prior resistant Gram-positive organism. For pneumonia, it considers a history of MRSA infection/colonization in lungs, IV antibiotic use in the last 90 days, hospitalization of 2 or more days in the previous 90 days, or mechanical ventilation. For skin and soft tissue infection, it considers if the wound is purulent, evidence of necrotizing infection, or concern for orbital/periorbital cellulitis. For urinary tract infection, it considers a strong suspicion for ampicillin-resistant enterococcal UTI with an inability to use oral options. If empiric IV vancomycin is not appropriate, it suggests stopping empiric vancomycin for suspected MRSA or ampicillin-resistant enterococcal infections if these organisms are not recovered in cultures in 48-72 hours.

Navigate back to Figure 1..

## Table 1. Long description

A table categorizing appropriate and possibly appropriate indications for empiric vancomycin use. The table has two main sections: Appropriate empiric indications and Possibly appropriate indications if additional criteria met. The first section lists conditions such as bacterial meningitis, infective endocarditis, bone and joint infections, sepsis, catheter-related bloodstream infections, and septic shock. The second section includes conditions like febrile neutropenia, intraabdominal infection, pneumonia, skin and soft-tissue infection, and urinary tract infection, each with specific criteria that must be met. Each row lists a specific condition or infection, and the columns categorize these conditions based on the appropriateness of empiric vancomycin use.

Navigate back to Table 1..

## Table 2. Long description

A table comparing baseline characteristics of preintervention and postintervention groups. The table has 12 rows and 4 columns. Column headers are Characteristic, Preintervention group (n = 100), Postintervention group (n = 100), and P-value. Row labels include Age in years, median (IQR), Male gender, n (%), History of MRSA infection, n (%), Recent hospitalization, n (%), ICU during admission, n (%), and Ordering indication, n (%). Row 1: Age in years, median (IQR), 65 (53–76), 66 (54–75), 0.915. Row 2: Male gender, n (%), 45, 44, 1.000. Row 3: History of MRSA infection, n (%), 7, 7, 1.000. Row 4: Recent hospitalization, n (%), 40, 34, 0.464. Row 5: ICU during admission, n (%), 28, 35, 0.361. Row 6: Ordering indication, n (%), BSI, 0, 0, 1.000. Row 7: BJI, 9, 23, 0.011. Row 8: CNS, 0, 5, 0.059. Row 9: FN, 1, 1, 1.000. Row 10: IE, 1, 0, 1.000. Row 11: IAI, 1, 0, 1.000. Row 12: PNA, 29, 23, 0.420. Row 13: Sepsis/septic shock, 25, 29, 0.641. Row 14: SSTI, 32, 19, 0.051. Row 15: UTI, 1, 0, 1.000. Row 16: Other, 1, 0, 1.000.

Navigate back to Table 2..

## Table 3. Long description

A table comparing outcomes of preintervention and postintervention groups in a study evaluating IV vancomycin therapy. The table has 13 rows and 4 columns. The columns are labeled Outcome, Preintervention group (n=100), Postintervention group (n=100), and P-value. The rows are labeled with various outcomes and their respective values for both groups, along with the P-value indicating statistical significance. Row 1: Appropriate order, n (percent), Preintervention group 55 (55), Postintervention group 97 (97), P-value less than .001. Row 2: Days of inpatient vancomycin therapy, n (IQR), Preintervention group 4 (3-6), Postintervention group 4 (2-7), P-value .609. Row 3: Outcome of empiric therapy, Continued as definitive therapy, Preintervention group 3, Postintervention group 8, P-value .221. Row 4: Continued empirically-appropriate, Preintervention group 15, Postintervention group 8, P-value .154. Row 5: Continued empirically-not appropriate, Preintervention group 22, Postintervention group 31, P-value .177. Row 6: Stopped by 48-72 hours, Preintervention group 60, Postintervention group 50, P-value .169. Row 7: Switched to alternative IV anti-MRSA drug, Preintervention group 0, Postintervention group 3, P-value .083. Row 8: AKI, Preintervention group 17, Postintervention group 22, P-value .476. Row 9: Infusion reaction, Preintervention group 3, Postintervention group 0, P-value .246. Row 10: 30-day mortality, Preintervention group 8, Postintervention group 11, P-value .631. Row 11: DOT per 1,000 patient days, Preintervention period 69.3, Postintervention period 64.2, P-value less than .041. Row 12: Vancomycin levels ordered per 1,000 patient days, Preintervention period 52.3, Postintervention period 31.1, P-value less than .001.

Navigate back to Table 3..
